# Selective multiple analyte detection using multi-mode excitation of a MEMS resonator

**DOI:** 10.1038/s41598-022-09365-9

**Published:** 2022-03-28

**Authors:** Usman Yaqoob, Nizar Jaber, Nouha Alcheikh, Mohammad I. Younis

**Affiliations:** 1 Physical Science and Engineering Division, King Abdullah University of Science and Technology, Thuwal, Saudi Arabia; 2 Department of Mechanical Engineering and the Center for Communication Systems and Sensing, King Fahd University of Petroleum and Minerals, Dhahran, 31261 Saudi Arabia; 3 Department of Mechanical Engineering, State University of New York at Binghamton, Binghamton, NY 13902 USA

**Keywords:** Sensors and biosensors, Mechanical and structural properties and devices

## Abstract

This work reports highly selective multiple analyte detection by exploiting two different mechanisms; absorption and thermal conductivity using a single MEMS device. To illustrate the concept, we utilize a resonator composed of a clamped-guided arch beam connected to a flexural beam and a T-shaped moveable mass. A finite element model is used to study the mode shapes and mechanical behavior of the device with good agreement reported with the experimental data. The resonator displays two distinct out-of-plane modes of vibration. For humidity detection, we utilize physisorption by functionalizing the surface with graphene oxide (GO), which has strong affinity toward water vapors. The GO solution is prepared and drop-casted over the mass surface using an inkjet printer. On the other hand, cooling the heated flexural beams is used for helium (He) detection (thermal-conductivity-based sensor). The sensor characteristics are extensively studied when the modes are individually and simultaneously actuated. Results affirm the successful utilization of each mode for selective detection of relative humidity and He. This novel mode-dependent selective detection of multiple analytes can be a promising building block for the development of miniature, low-powered, and selective smart sensors for modern portable electronic devices.

## Introduction

Recently, the development of high-performance gas/chemical sensors with the ability to selectively detect a target analyte has seen increasing attention for its realization in various fields including environmental monitoring, medical diagnostics, and industrial product manufacturing^1,2^. Among the several reported mechanisms, metal oxide semiconductor (MOS) chemiresistive sensors were extensively studied and implemented as commercial products for the detection of different gases/chemicals due to their high sensitivity, fast response, easy fabrication, compact size, and simple measuring techniques^3^. However, due to the relatively high operating temperature, they tend to show response towards unwanted analytes having similar physicochemical properties thus suffer from poor selectivity^4–6^. As of now, different sensing material development strategies are implemented to enhance sensors performance including constructing different nanostructures^7,8^, development of composition/hybrids, hetero/homo-junction formations^9–14^, and surface engineering through dopants^15^. These techniques certainly enhance the sensor performance in various aspects such as sensitivity, fast response/recovery time, and wide detection range at room temperature. However, selective analyte detection under humid/dry conditions with good repeatability and reproducibility is still an open challenge. Filtering unwanted gases/chemicals has been used to resolve the selectivity issue in which gases/chemical mixtures are pre-separated via a filter membrane having a specific pore size to allow the target analyte to pass through and reach the sensor surface. Materials having microporous surfaces such as zeolite, graphene, polymers, metal–organic frameworks can be widely tuned according to the target analyte size; and thus are considered promising candidates^16,17^. Nevertheless, these filter membranes might be restricted to a very small size target analyte as the bigger pores may allow diffusion of several other gases/chemicals with similar diameters resulting in cross-sensitivity^16^. Alternatively, electronic nose systems (E-nose) based on arrays of sensors have been used to significantly improve the selectivity through signal processing (features extraction) and subsequent implementation of pattern recognition algorithms for the model training^18,19^. However, they are complex, expensive, consume high power, and require a large hardware setup for their implementation in a realistic application. Therefore, there is an urgent need to build alternative strategies for the development of smart low-powered tiny sensors with excellent selectivity and stability^20^.

Micro and nanoelectromechanical systems (MEMS and NEMS) resonators show high potential in detecting physical/chemical stimuli such as mass quantification, pressure sensing, and gas detection due to their trace level detection ability, miniaturize structure, batch fabrication process, and low power consumption^21–24^. Several dynamics-based mechanisms of electrostatically actuated resonators have been explored to enhance sensing, such as bifurcation points, buckling points, weakly coupled resonators, and the coupling between bending and torsional modes^25–29^. However, most of these resonators possess low-quality factors; thus a low-pressure environment is required to obtain satisfactory results^30^.

The coating of selective materials on the resonator surface is essential for the adsorption of a particular analyte to significantly improve the sensitivity and selectivity of the device^31,32^. Particularly, for humidity detection, various methods have been used including surface acoustic waves (SAW), quartz crystal membranes (QCM), and MEMS resonators, which are coated with different materials such as graphene oxide, metal–organic frameworks (MOF), and polymers composite^33–36^. In our previous work^37^, we showed, based on heated buckled micro-beam, a selective identification for some gases without the need for the material coating. These sensors are based on changing the thermal energy dissipation (cooling or heating) of the micro-beam in the presence of a certain gas concentration^37^. Also, we showed an attractive way in detecting and categorizing different gases according to their thermal conductivity based on simultaneously tracking the frequency response of multiple modes of vibration of micro-resonator operated near the buckling zone^37^.

The simultaneous actuation of multiple vibrational modes opens up new frontiers for the development of highly selective mode-dependent detection of multiple gases/chemicals using a single resonator structure. The design of a resonator with the ability to simultaneously actuate multiple and distinct vibrational modes is critical to utilize each mode for the detection of a specific gas^38,40^.

Herein, we propose selective detection of gas species based on two modes of sensing. As a case study, we target humidity and helium (He) by utilizing distinct vibration modes of a clamped-guided arch beam connected with flexural beams and T-shaped moveable mass. One mode of vibration is dominant by the moveable mass motion and the other by the flexural beams bending. The moveable mass surface coated with graphene oxide (GO) is utilized for humidity detection, while the thermally heated flexural beams are used for He detection. The GO shows high affinity towards water vapors due to the existence of different oxygen functional groups on its surface and edges. On the other hand, the axially stressed flexural beams become very sensitive to the surrounding gas heat capacitance and shows an increase in stiffness after He exposure. The proposed muti-sensor is a promising candidate to be utilized for simultaneous gas/chemical monitoring applications.

## Device design, operation, and experimental arrangements

**Figure 1:**
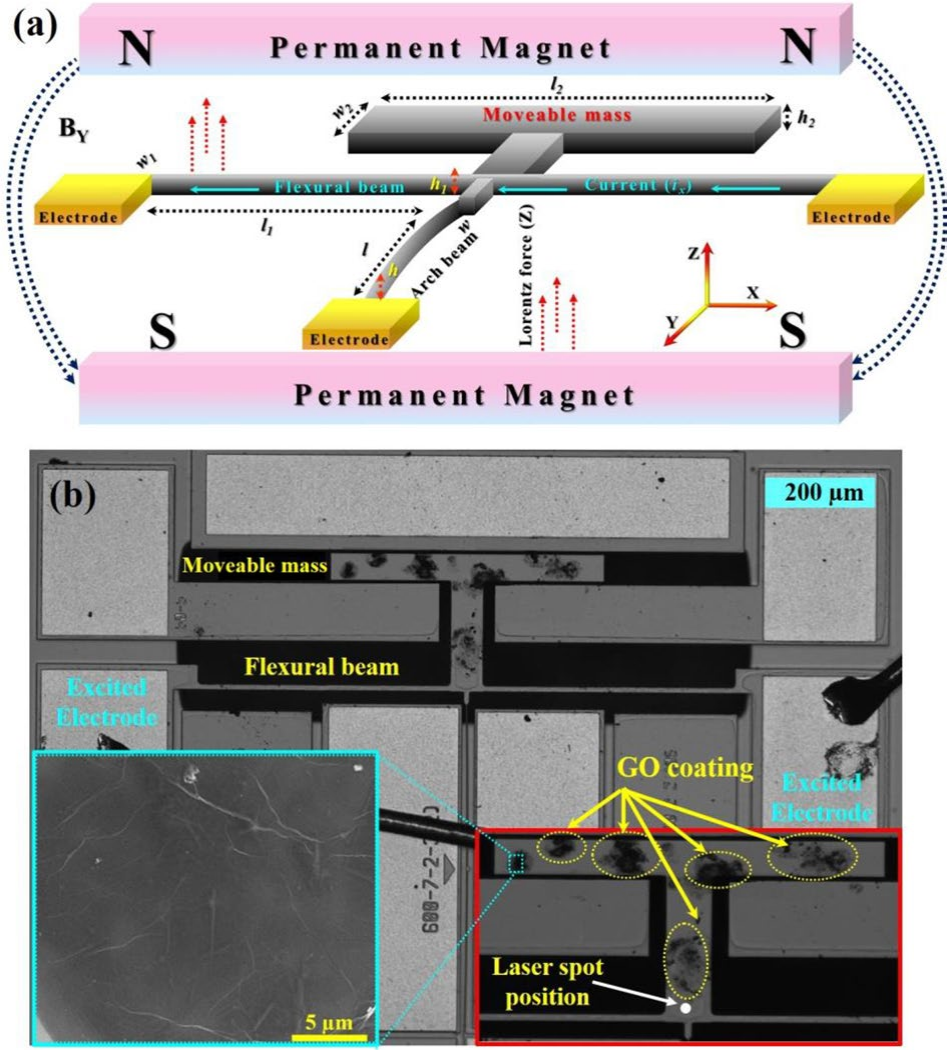
Device structure, (**a**) a schematic illustration of the device dimensions and setup for electro-magnetic actuation, (**b**) an optical image of the whole device. The inset shows a magnified image of the moveable mass. An SEM image (highlighted with light blue) is also shown to indicate the homogeneous distribution of GO sheet on the moveable mass surface.

An in-plane multiple resonance microstructure was fabricated by MEMSCAP using a silicon-on-insulator (SOI) wafer with a device layer of highly conductive n-doped silicon. Our device is composed of a clamped-guided arch beam (length (*l*) = 600 µm, width (*w*) = 1.7 µm, height (*h*) = 25 µm, and initial rise (arch beam curvature: *b*_*o*_ = 2.5 µm) connected with two flexural beams (each of dimensions *l*_*1*_ = 460 µm, *w*_*1*_ = 10 µm, *h*_*1*_ = 25 µm) and a T-shaped moveable mass (*l*_*2*_ = 500 µm, *w*_*2*_ = 50 µm, *h*_*2*_ = 25 µm). The device was electro-thermally and electro-magnetically actuated by applying a small thermal voltage (V_th_) to flexural beams in the presence of a permanent magnetic field of ~ 100 mT to generate an out-of-plane Lorentz force. Figure 1 shows a schematic for the actuation of the out-of-plane vibration modes with a microscopic image of the real device.

**Figure 2:**
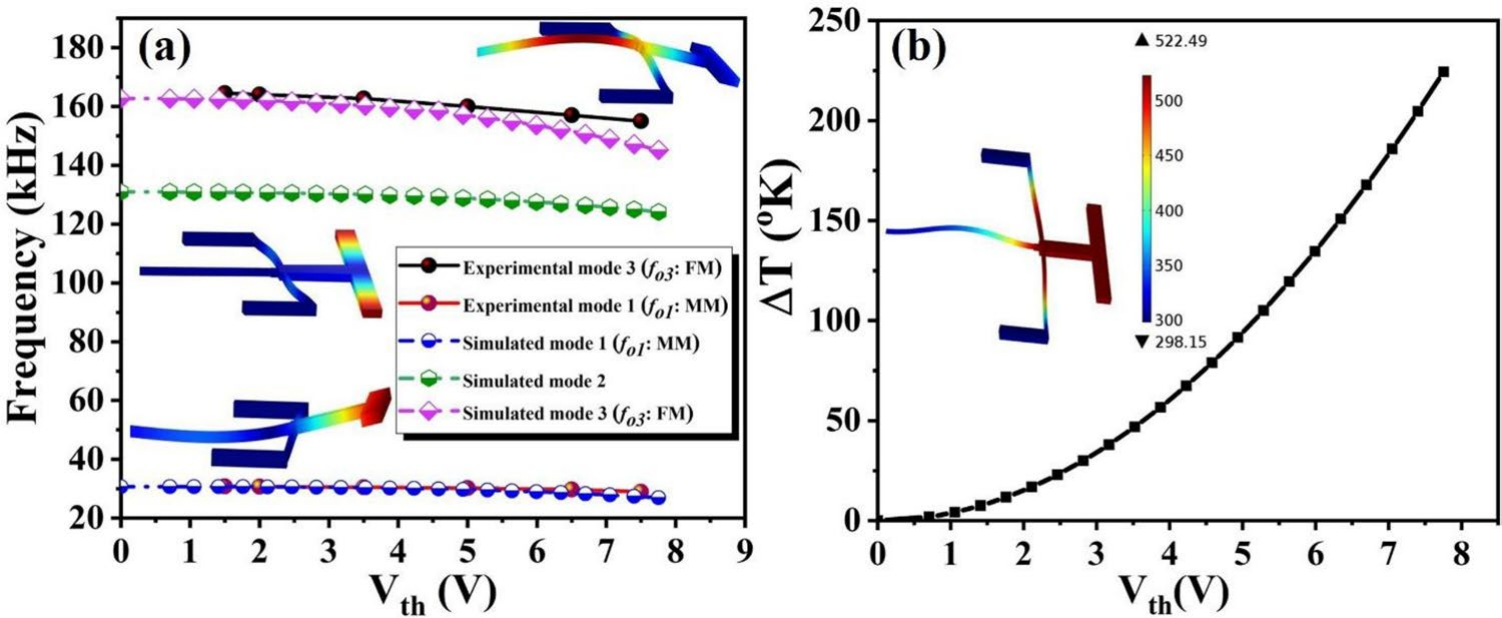
Experimental and finite-element results for an uncoated device. (**a**) Experimental data and simulated results for the variation of the resonance frequency of the first three out-of-plane modes under electro-thermal actuation (V_th_). Insets show their corresponding mode shapes. (**b**) Simulated results for the variaton of the temperature at the centre of the flexural beam under V_th_. Inset shows the temperature distrubation of the device at V_th_ = 7.7 V.

A finite-element (FE) model of the device was built with the COMSOL software to design the devices and to simulate their natural frequencies with mode shapes under electro-thermal voltage. A 3-D multi-physics simulation was conducted by coupling the Heat Transfer, Solid Mechanics, and Electric Currents interfaces^37–41^. Applying V_th_ into the flexural micro-beams creates a compressive stress, which affects the resonance frequency of the device. Figure 2a shows the experimental data and the simulated results for the first three out-of-plane modes natural frequencies under electro-thermal voltage actuation (V_th_). As we can see, the results show a good agreement between them. As shown in Fig. 2a, V_th_ changes the flexural micro-beams stiffness resulting into a decrease in its resonance frequencies.

In addition, the insets schematic in Fig. 2a indicates that the local motion of the moveable mass dominates in mode 1 [mass mode (MM)] and the local motion of the flexural micro-beam dominates in mode 3 [flexural mode (FM)]. To wwfurther study the effect of V_th_ on the temperature distribution of the device, we extract and plot the variation of temperature under different V_th_, see Fig. 2b. As seen, when V_th_ increases, the temperature increases until it reaches a maximum value of 522°K at 7.7 V, which is much lower than the melting point of silicon, 1687°K. As shown in the inset of Fig. 2b, the central area of the flexural micro-beam has the highest temperature, which decreases gradually toward the anchors.

### Graphene oxide preparation and deposition on the MEMS device

The single-layer graphene oxide (GO) powder (CN: NC1116347) from Thermo Fisher Scientific was used without further purification. A highly dispersed GO solution using MilliQ water was prepared through vigorous ultra-sonication in a water bath tank for 30–45 min at room temperature. Then, as-synthesized GO solution was transferred to the 2 ml reservoir tube of an inkjet printer for deposition. MicroFab jetlab II printing platform with a jetting device of 40 µm was used to precisely deposit the GO droplets over the T-shaped moveable mass surface. A magnified microscopic image with the encircled GO droplet areas is shown in the inset of Fig. 1b.

### Characterizations and sensing setup

The structural defects and chemical properties of GO were acquired through the WItec Apyron Raman system (spectral range 700–3500 cm^−1^) and Nicolet iS50 Fourier transforms-infrared spectroscopy (FT-IR) (spectral range 700–3800 cm^−1^). A Quanta 3D FEG electron scanning microscopy (SEM) was used to observe the surface morphology and the nature of the deposited GO contents on the moveable mass surface.

The gas sensing characterizations were carried out at room temperature under atmospheric pressure in synthetic air. In all measurements, a constant flow rate of gases mixture was maintained at 2 SLPM (standard liters per minute) through a computerized mass flow control system (Alicat Scientific). The Thermo Scientific VersaCool chiller was used to maintain the bubbler temperature for water vapors generation. Nitrogen (N_2_) was used as a carrier gas and the water vapors were generated under a constant bubbler temperature of 21 °C. The maximum water vapors concentration was calculated around 24,462.9 ppm in N_2_ (carrier gas) at 21 °C, using Eqs. (1) and (2) below, which was considered as approximately equal to 100% RH when directly supplied to the test chamber^23^:

1 $${Log}_{10}{P}_{v}=A-\frac{B}{C+T},$$

where ${P}_{v}$ is the partial pressure of generated vapors, *T* is the bubbler temperature, *A* = 8.071,* B* = 1730.63, and *C* = 233.426 are the constants used for the calculation at 21 °C.

2 $${C}_{v}=\frac{{P}_{v}}{{P}_{atm}}\times {10}^{6},$$

where ${C}_{v}$ is calculated vapors concentration in ppm, and ${P}_{atm}$ is atmosphereic pressure.

Further, different levels of RH and He gas were achieved by varying the flow rate of the target analyte (RH/He) and dry N_2_ gas in the test chamber using Eq. (3):

3 $$Target \; concen.=\frac{flowrate \; of \; target \; analyte}{flowrate \; of \; target \; analyte +{flowrate \; of \; N}_{2}}\times Supplied \; gas \; concen. \; \left(\mathrm{ppm}\right).$$

**Figure 3:**
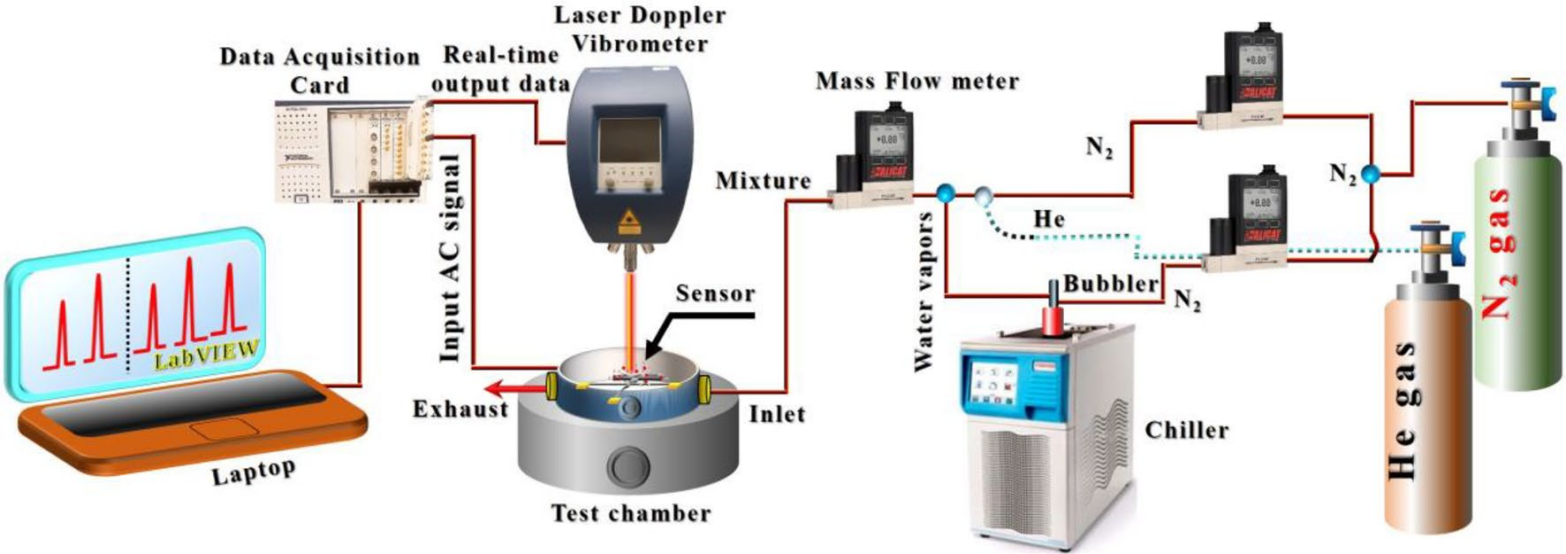
Schematic illustration of the gas sensing setup.

A laser Doppler vibrometer (MSA-500) was used to monitor the resonator response to determine the resonance frequencies, their corresponding mode shapes, and to track the shift in amplitude during the target analyte exposure. A data acquisition card (DAQ) was utilized to excite the flexural beams with different AC signals and then record the real-time output data from the vibrometer through the LabVIEW software. A schematic demonstration of the whole gas sensing setup is displayed in Fig. 3.

## Results and discussion

**Figure 4:**
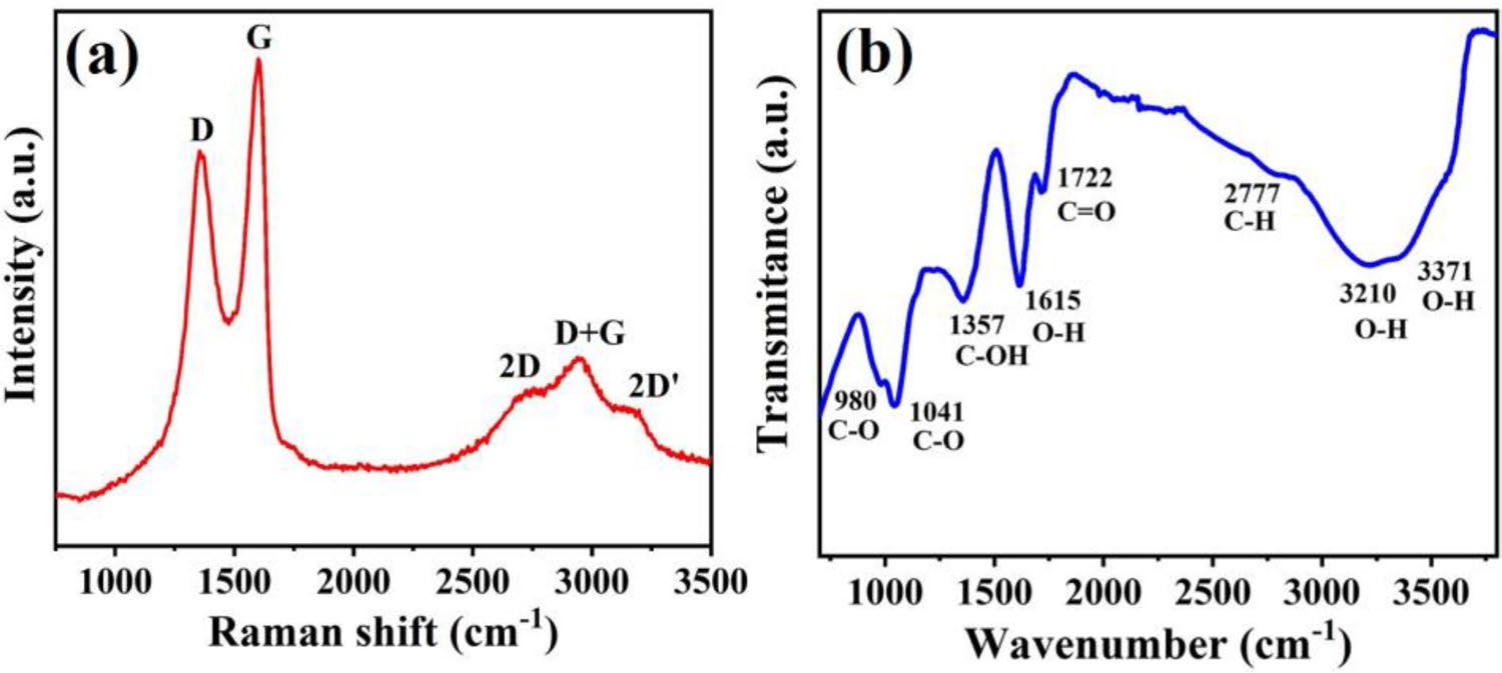
Graphene oxide characterizations, (**a**) Raman spectra, and (**b**) FT-IR analysis.

An SEM image was taken to observe the graphene oxide (GO) sheets distribution over the moveable mass surface. The homogeneous extension of the GO sheet with little crumples can be seen, revealing a high surface area for the water molecule adsorption (inset of Fig. 1b). Raman and FT-IR characterizations were carried out to determine the physical and chemical properties of the GO. Raman spectroscopy is widely used and considered a reliable tool to determine the vacancies and defects in the 2-D allotrope of carbon. The existence of the D (at 1355 cm^−1^), backscattered 2D (2717 cm^−1^), and 2D' (3187 cm^−1^) peaks confirms the structural disorder in the Sp^2^ arrangements of the benzene ring (Fig. 4a)^42^. Further, the FT-IR spectra was carried out to observe the existence of functional groups and their relative bending/stretching vibrations. Figure 4b presents the FT-IR spectra, which demonstrates clear and intense peaks related to different functional groups. A broadband peak appears near 3200 cm^−1^, which can be attributed to the stretching vibrations of O–H bond (hydroxylic). The stretching vibrations for carboxyl (C=O) was found at 1722 cm^−1^ and the existence of a sharp resonance peak at 1615 cm^−1^ can be attributed to the stretching/bending vibrations of the O–H groups from adsorbed water molecules. The peaks near 980 cm^−1^ and 1041 cm^−1^ can be assigned to the stretching/bending of epoxy (C–O) group^42^. The existence of defects and subsequent formation of various oxygen functional groups are responsible for the remarkable adsorption of water molecules on the GO surface. The details are discussed in the sensing mechanism section^43,44^.

**Figure 5:**
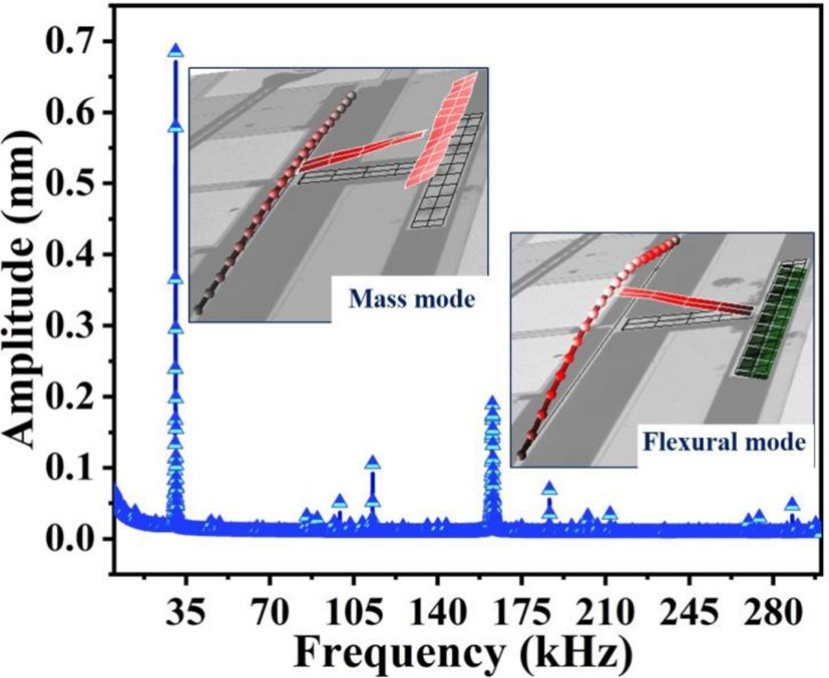
Frequency response due to white noise excitation with measured mode shapes.

Further, to explore the resonance modes, the device was excited with a white-nose signal (V_th_ = V_ac_ = 1.5 V) in the presence of ~ 100 mT magnetic field under atmospheric pressure in synthetic air. The two out-of-plane vibration modes appeared, in which the moveable mass mode (MM) dominates at *f*_*o1*_ = 30.66 kHz while the flexural beam mode (FM) dominates the motion at *f*_*o3* =_ 162.85 kHz. The white noise response of the device with the corresponding measured mode shape is shown in Fig. 5. The mass mode (MM @ 30.66 kHz) is utilized for relative humidity detection due to its higher surface area for comparatively easy graphene oxide deposition. On the other hand, the axially-stressed flexural beam mode (FM @ 162.85 kHz) is exploited for He detection due to its high sensitivity to the surrounding environment temperature changes.

### Frequency response and sensing performance of individually actuated modes

**Figure 6:**
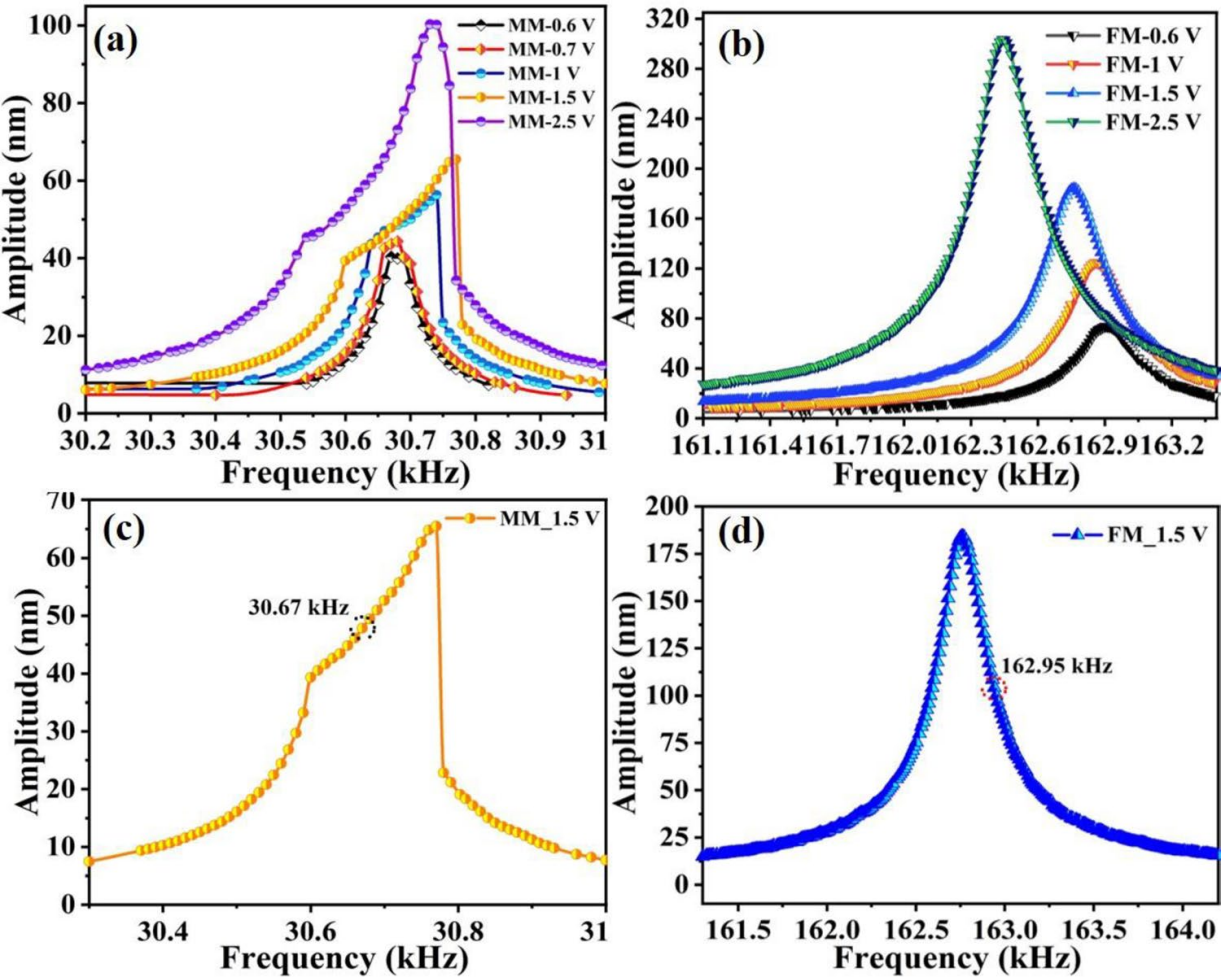
Frequency response curves for the resonator, (**a**) frequency sweep at mass mode under different operating voltages, (**b**) frequency sweep at flexural mode under different operating voltages, (**c**) mass mode frequency response obtained at V_ac_ = 1.5 V encircled with the selected single frequency for actuation, and (**d**) flexural mode frequency response obtained at V_ac_ = 1.5 V encircled with the selected single frequency for actuation.

To record the maximum resonance amplitudes of each mode, the measurements were carried out after fixing the laser spot at the center of the junction point of the moveable mass and flexural beams as depicted in the inset of Fig. 1b. Then, the frequency response curves were generated by sweeping the frequency of the electro-thermal AC source with different magnitudes near the mode of interest. The frequency response for the mass mode at different AC loadings is shown in Fig. 6a. An increase in the resonance amplitude and negligible shift in frequency was observed with the increase in V_ac_ values. No significant shift in frequency suggesting minimal effect of heated flexural beams on the mass mode. On the other hand, the frequency response at the flexural mode reveals a linear response with the shift in amplitude as well as in frequency under all applied conditions (Fig. 6b). The shift in frequency at different AC signals can be attributed to the DC effect of the AC signals in the directly heated flexural beams, which causes compressive axial load that reduces the flexural beams stiffness. The frequency response curves attained after frequency sweeping at V_ac_ = 1.5 V (power (P) = 2.32 mW) near the resonance of each vibration modes were selected for further sensing characterizations. Figure 6c,d present the frequency response curves for each vibration mode with the encircled points referring to the operating frequency for each mode.

**Figure 7:**
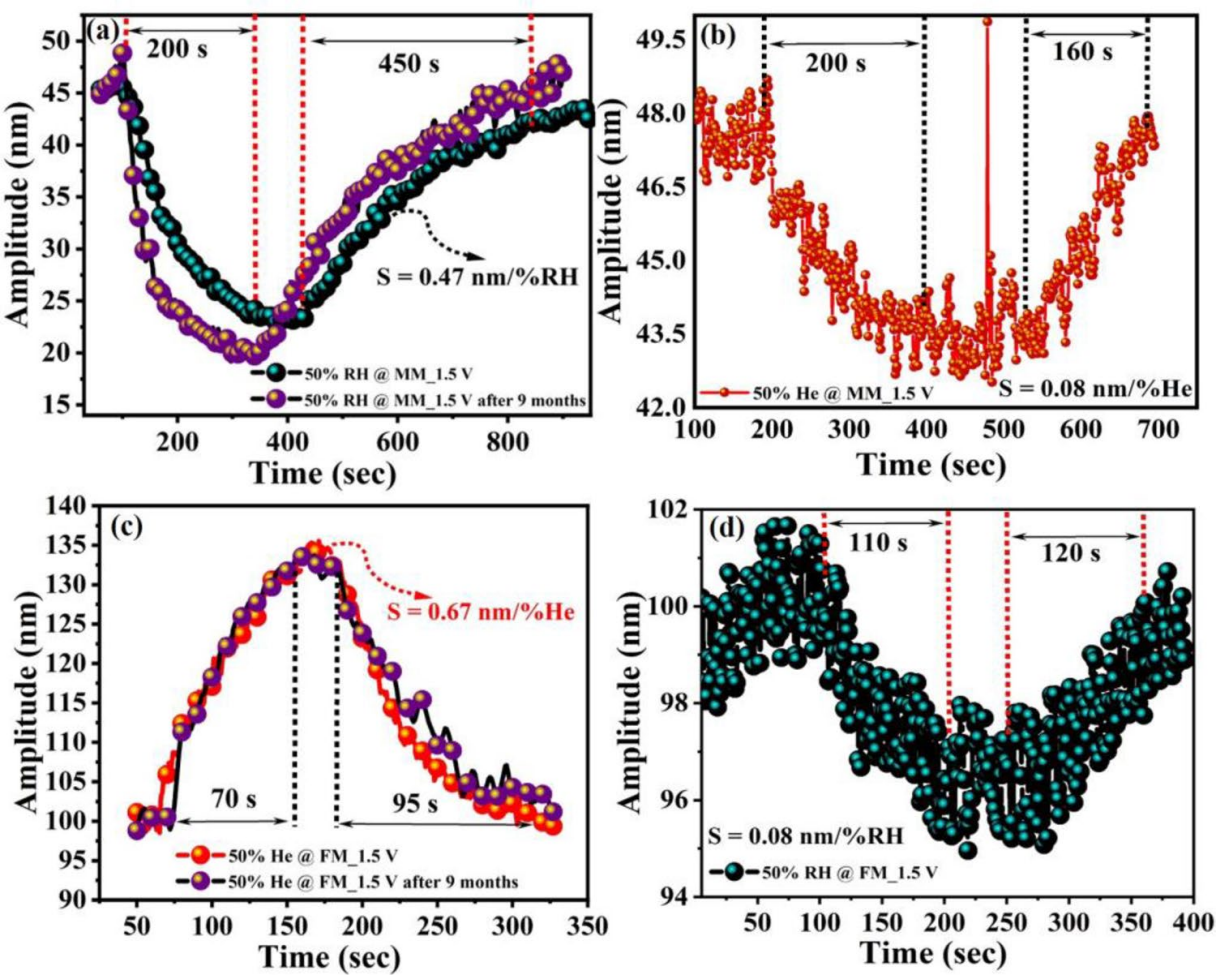
Sensing results of the individually actuated vibration modes, (**a**) responses to 50% relative humidity before and after nine months at mass mode operated at *f*_*o1*_ = 30.67 kHz, (**b**) response to 50% He at mass mode (MM) operated at *f*_*o1*_ = 30.67 kHz, (**c**) responses to 50% He before and after nine months operated at flexural mode operated at *f*_*o3*_ = 162.95 kHz, and (**d**) responses 50% relative humidity at flexural mode (FM) operated at *f*_*o3*_ = 162.95 kHz.

The sensing response was carried out after actuating the mode of interest at the selected single frequency as depicted in Fig. 6c,d Before target analyte exposure, the test chamber was continuously flushed with the dry N_2_ gas for at least 15–20 min to eliminate the humidity contents from the chamber and to generate synthetic air environment. Figure 7 shows the real-time change in resonance amplitude at both modes after 50% RH and 50% He exposure. The mass mode actuated at *f*_*o1*_ = 30.67 kHz, left side of the frequency response curve (Fig. 6c), demonstrates a strong response towards relative humidity, which can be attributed to the direct adsorption of water molecules on the GO surface. A decrease in amplitude was observed during the adsorption of water molecules on GO (Fig. 7a). The details on the working mechanism are discussed in the sensing mechanism section. Figure 7b shows a slight change at the mass mode upon He exposure, which can be attributed to the stiffness change of the heated flexural beams upon humidity exposure. Conversely, the flexural mode excited at *f*_*o3*_ = 162.95 kHz exhibits a higher response to He, which can be attributed to the higher sensitivity of flexural beams to the surrounding gas thermal conductivity (Fig. 7c). For the flexural mode actuation, the selected operating frequency point was on the right side of the frequency response (Fig. 6b). Therefore, an increase in amplitude during He exposure suggests stiffness increase of the beam, which is due to the higher thermal conductivity of He that cools down the beam^37^. The appearance of a slight change at the flexural mode in the presence of relative humidity might be due to the adsorption of water molecules on GO surface at the mass mode, which forces a change at the flexural mode (Fig. 7d). Moreover, the sensor performance in terms of sensitivity (*S*) and response/recovery time was also calculated and summarized in Fig. 7. *S* values were calculated using S = *A*_*b*_ − *A*_*g*_/Δ*analyte%,* where ${A}_{b}$ is the baseline amplitude, ${A}_{g}$ is the amplitude change after water vapors/gas molecule exposure, $\%analyte$ is percentage of target analyte (%RH or %He), and Δ*analyte%* is the change in target analyte percentage before and after its exposure to the chamber.

**Figure 8:**
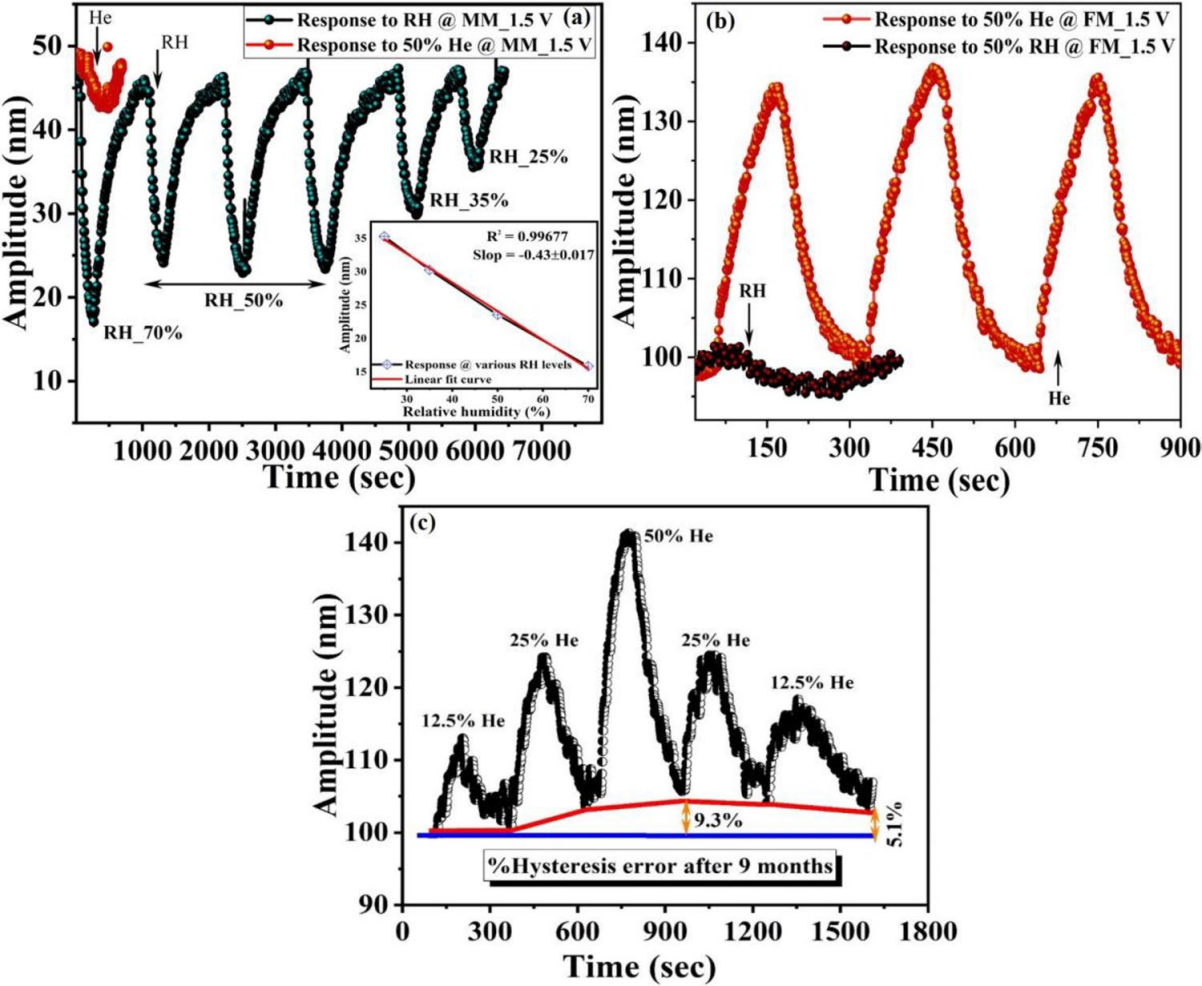
Linearity and repeatability test of the individually actuated vibration modes operated at V_ac_ = 1.5 V, and *f*_*o3*_ = 162.95 kHz (flexural mode), (**a**) response of MM operated at *f*_*o1*_ = 30.67 kHz to different levels of relative humidity with repeatability (single-cycle response of 50% He is also attached), (**b**) repeatability graph for 50% He at FM operated at *f*_*o3*_ = 162.95 kHz. For comparison, a single-cycle response to 50% relative humidity is also shown in the graph, and (**c**) response to different concentration of He at FM after nine months showing the hysteresis test results.

Figure 7a,b show mass mode response results to RH and He with the calculated *S* of 0.47 nm/%RH and 0.08 nm/%He, respectively. In addition, the results show a response/recovery time of 200 s/450 s for RH and 200 s/160 s for He. Hence, the results demonstrate that operating at the mass mode (MM), the proposed sensor has high sensitivity towards Humidity. Similarly, Fig. 7c,d summarize the sensing results and the time responses of flexural mode (FM) for 50% He and 50% RH, where the micro-sensor becomes very sensitive to He with a maximum *S* of 0.67 nm/%He and a response/recovery time of 70 s/95 s. The results suggest successful utilization of the two vibration modes MM and FM for selective detection of relative humidity and He. Moreover, to test the stability of the proposed microsensor, the device response was tested after nine months, see Fig. 8a,c. As we can see, the results show a small shift in the sensor response, which demonstrates good stability of the device.

To test the repeatability of the device, the sensor response was examined at MM and FM under different relative humidity and He levels, see Fig. 8a,b. The multi-sensor demonstrates a good repeatability and excellent linearity (inset of Fig. 8a). In addition to the good repeatability and linearity, low hysteresis effect is one of the performance metrics for the gas sensing. Hence, the response of He sensing was performed after nine months and at different concentration levels, Fig. 8c. The results reveal excellent stability and good linearity. However, it was noticed that the resonator did not reach the baseline, and the hysteresis error for 50% He was calculated around 9.3%. This error reduced to 5% when the concentration value reached 12.5% (Fig. 8c).

**Figure 9:**
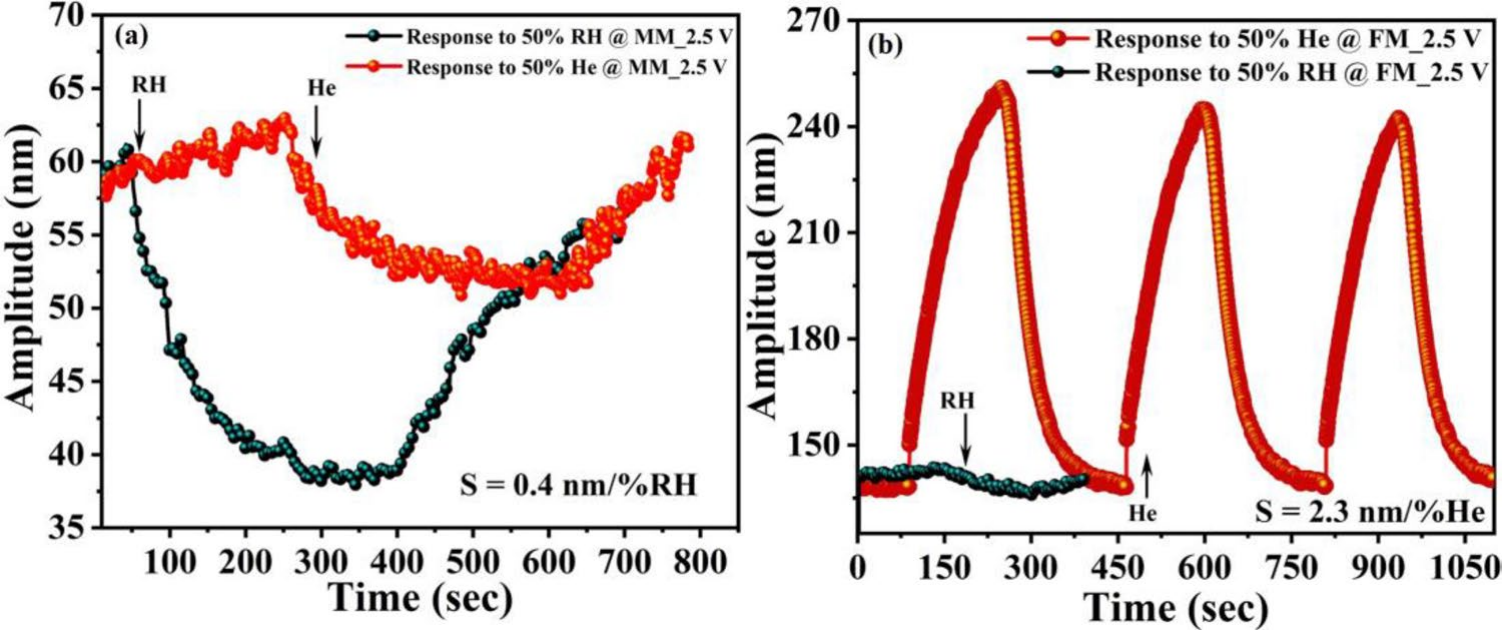
Response of individually actuated vibration modes when excited with V_ac_ = 2.5 V, (**a**) response to 50% RH and 50 He% (single cycle) at MM, (**b**) response to 50% He with repeatability and 50 He% single cycle at FM.

Moreover, it was found that the operating voltages has significant effect on the sensing performance of the resonator. The sensor response at an applied voltage of V_ac_ = 2.5 V was checked and shown in Fig. 9. As expected, a significant increase in He detection at FM and a drastic decrease in relative humidity detection at MM was found as depicted in Fig. 9. The drastic decrease in humidity detection can be attributed to the elevated temperature of the structure at higher voltage, which hinders the absorption ability of the coating material.

### Frequency response and sensing performance of simultaneously actuated modes

**Figure 10:**
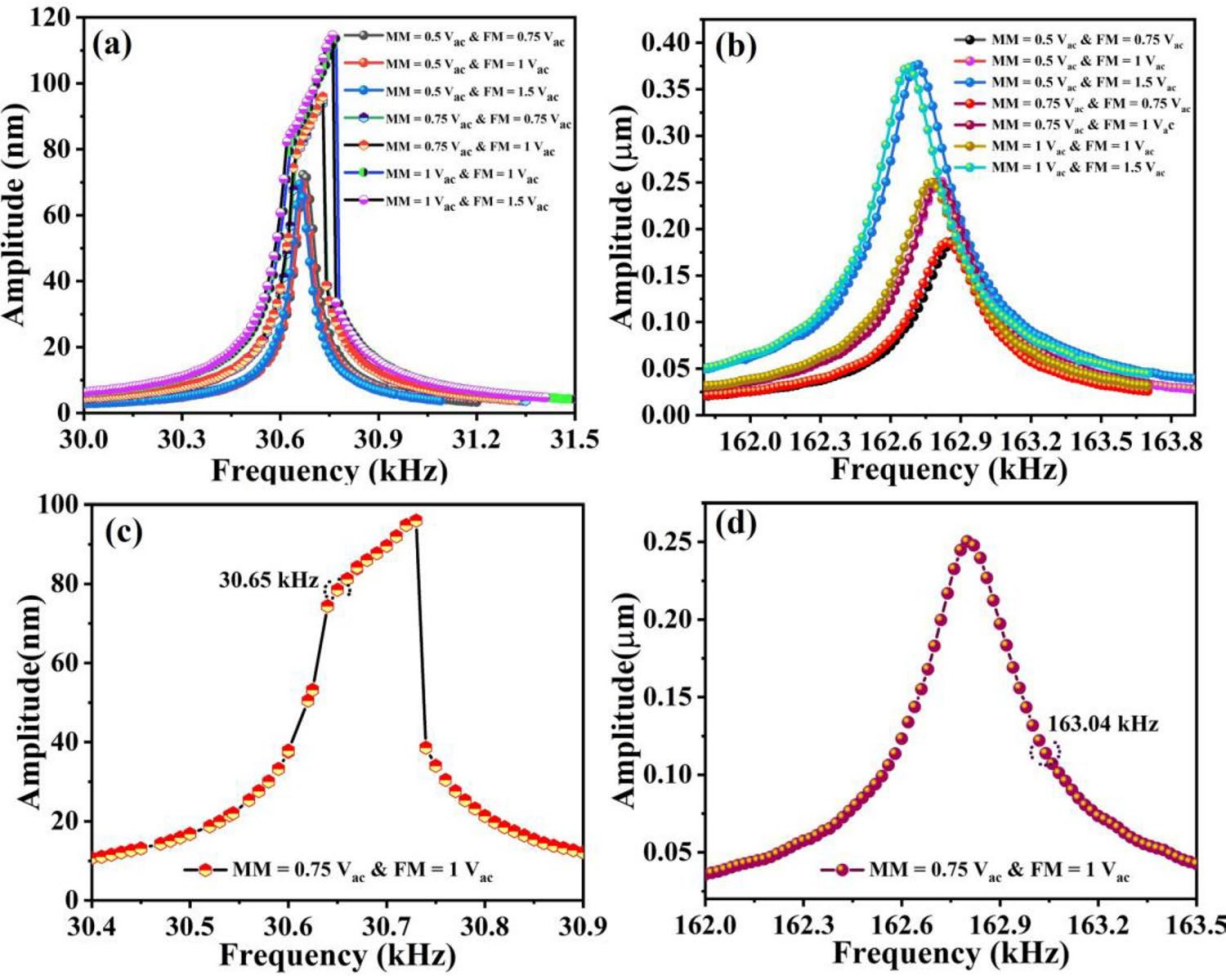
Frequency response curves obtained under various combinations of V_ac_, when both modes are simultaneously actuated, (**a**) frequency sweeping curves at the mass mode while the flexural mode was constantly actuated with a single frequency near its resonance, (**b**) frequency sweeping curves at the flexural mode while the mass mode was constantly actuated with a single frequency near its resonance, (**c**) selected frequency response curve for gas sensing, obtained after sweeping at the mass mode when the flexural mode constantly actuated with a single frequency (operating conditions V_ac_ = 0.75 V, frequency range 30.4–30.9 kHz at the mass mode, V_ac_ = 1 V, single frequency: 163.04 kHz at flexural mode), and (**d**) selected frequency response curve for gas sensing, achieved by sweeping near flexural mode while the mass mode was constantly excited with a single frequency (operating conditions: V_ac_ = 0.75 V, single frequency: 30.65 kHz at the mass mode, V_ac_ = 1 V, frequency range 162–163.5 kHz at the flexural mode).

The sensing properties of the device are further investigated when both vibration modes are simultaneously actuated at their corresponding resonance frequency, which resembles the practical operating condition. In this case, the frequency response curve is obtained through frequency sweeping near the resonance of the desired mode while the other mode is constantly actuated at a single frequency near its resonance. The frequency response curves for both modes obtained under different combinations of excitation voltages (V_ac_) are shown in Fig. 10a,b. At mass mode, an increase in amplitude with a slight shift in frequency was observed at the higher V_ac_ values (Fig. 10a). The negligible shift in frequency suggests less heat dissipation at the moveable mass surface. On the other hand, frequency sweeping near the flexural mode at higher V_ac_ values reveals an increase in amplitude as well as shift in frequency. In this case, frequency shifts to lower values, which can be attributed to the flexural beams stiffness degradation due to the direct implementation of the axial load (Fig. 10b). The frequency response curves for both modes obtained under the operating conditions of V_ac_ = 0.75 V at the mass mode and V_ac_ = 1 V at the flexural mode were used for sensing investigations. Figure 10c,d shows the selected simultaneously actuated frequency response curves for both vibration modes (MM: *f*_*o1*_ = 30.65 kHz at V_ac_ = 0.75 V and FM: *f*_*o3*_ = 163.04 kHz at V_ac_ = 1 V) excited with a total input power of 3.16 mW.

**Figure 11:**
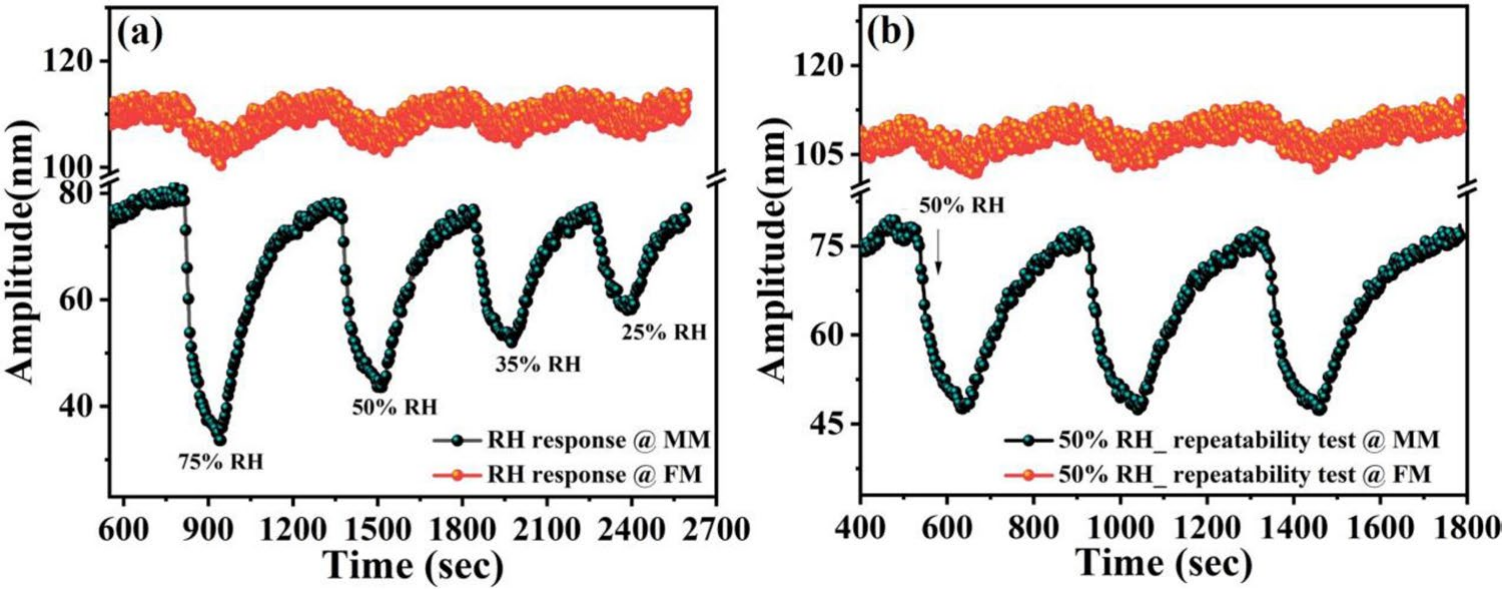
Response of simultaneously actuated vibration modes (V_ac_ = 0.75 V, *f*_*o1*_ 30.65 kHz at MM and V_ac_ = 1 V, *f*_*o3*_ 163.04 kHz at FM), (**a**) to different levels of relative humidity, and (**b**) simultaneous repeatability test for 50% relative humidity.

**Figure 12:**
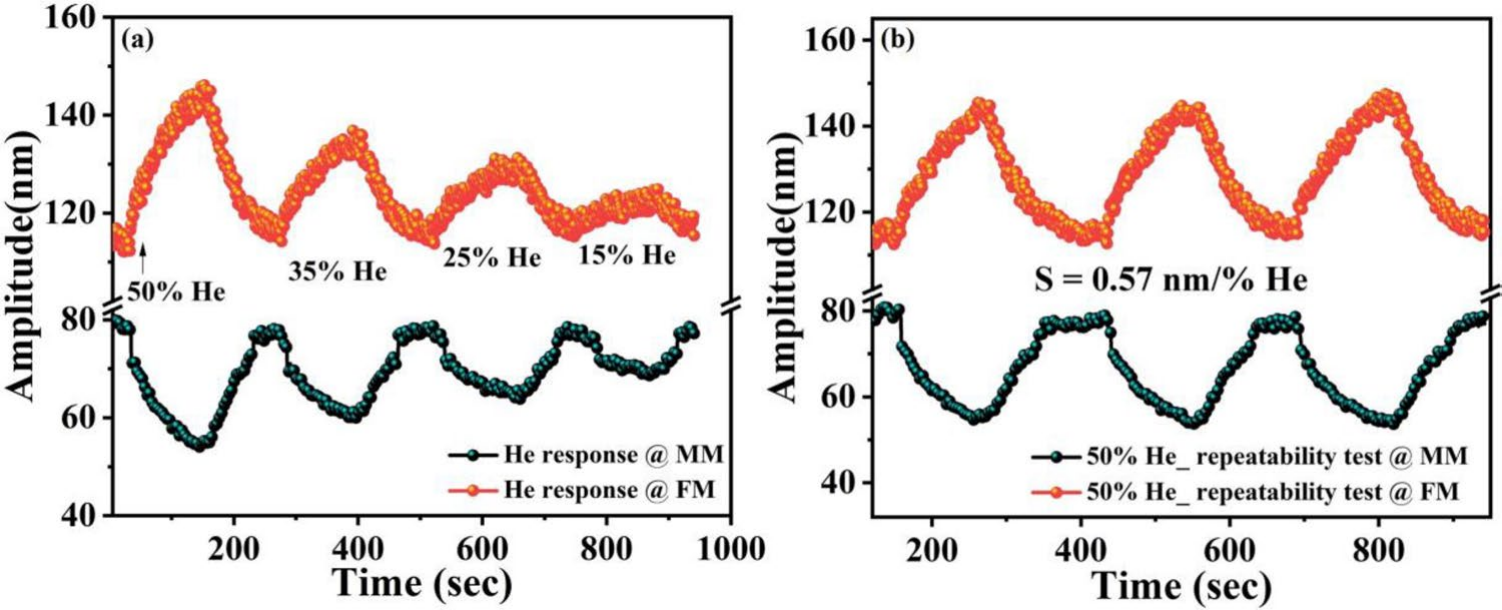
Response of simultaneously actuated vibration modes (V_ac_ = 0.75 V, *f*_*o1*_ 30.65 kHz at mass mode MM and V_ac_ = 1 V, *f*_*o3*_ 163.04 kHz at flexture mode FM), (**a**) simultaneous response of both vibration modes to different levels of He, (**b**) simultaneous response of both vibration modes to 50% He with repeatability.

Figure 11a shows the response of the simultaneously actuated modes under different levels of relative humidity. The simultaneous repeatability test at both modes to 50% RH were also performed and shown in Fig. 11b. On the other hand, during He exposure surprisingly a symmetric change in the amplitude of both modes was observed (Fig. 12). From the sensing results of the individually actuated modes, it is clear that He does not have an affinity towards graphene oxide at the mass mode. Therefore, a response at the mass mode upon He exposure can be attributed to the coolness of the flexural beams, which forces symmetric change due to the simultaneous actuation. The sensor response to different levels of He with repeatability test under 50% He is shown in Fig. 12b.

**Figure 13:**
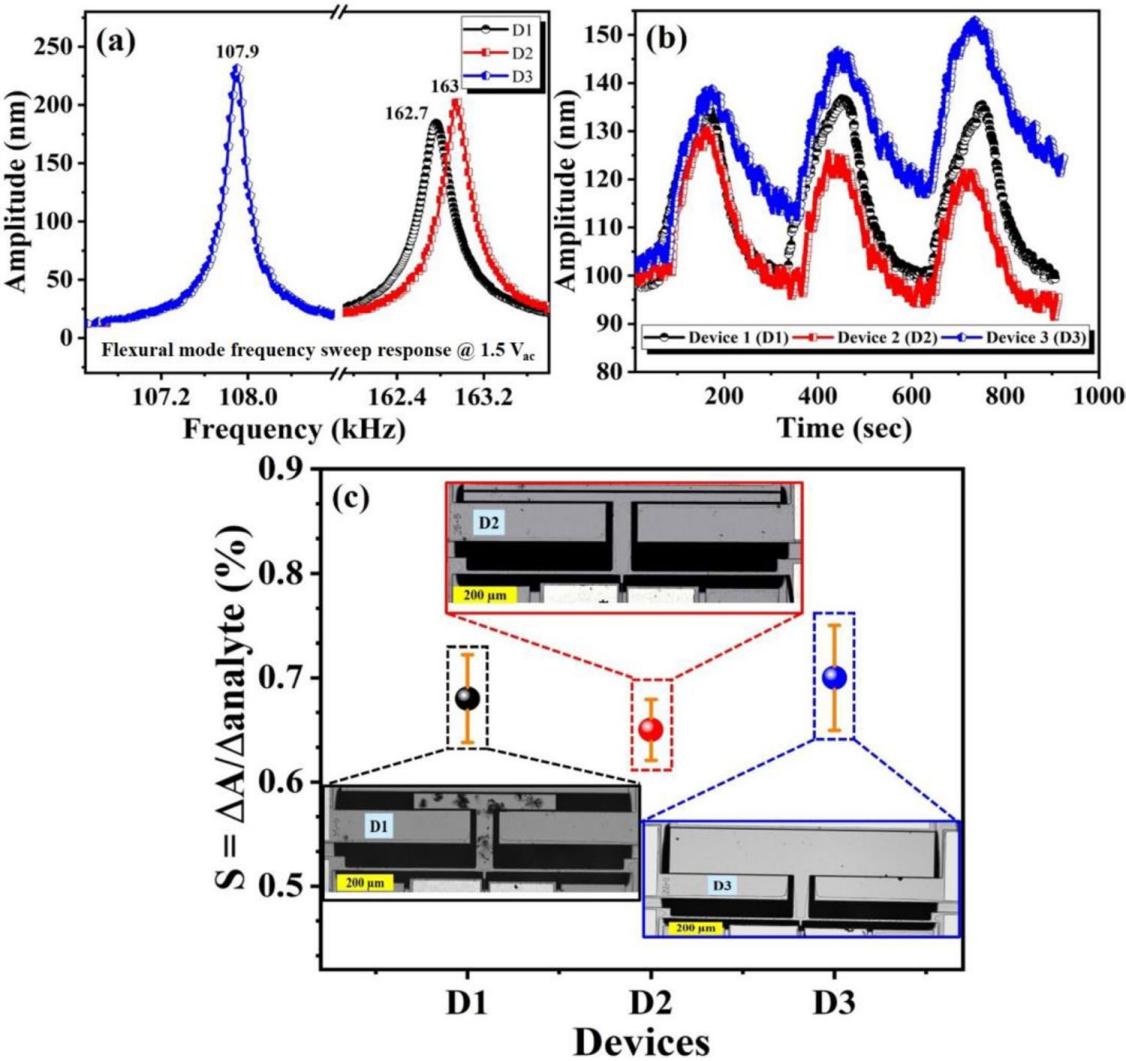
Dynamic and sensing characterizations of all three devices (D1,D2, and D3), (**a**) frequency sweep response, (**b**) 50% He response at flexural mode, (**c**) sensitivity results with error bars for D1, D2, and D3. Insets show the microscopic images of the devices.

It is worth mentioning that the sensitivity of the proposed sensor is strongly dependent on the microbeam stiffness (resonance frequency of the resonator and its mass).Therefore, different geometries of resonator will affect the sensitivity of the device. Particularly, for the absorption-based mode, the sensitivity S ∝ frequency/mass. Therefore, the geometrical parameters of the device will have an impact on its sensitivity, and thus, its performance. Hence, shrinking the device size will lead to a higher resonance frequency, and thus higher sensitivity. However, minimizing the big mass surface will reduce the surface sensing area. Hence, the geometrical parameters of the device should be optimized for high performance gas sensing. Moreover, we investigated experimentally the influence of T-shaped moveable mass surface on the flexural mode resonant frequency (FM) and the sensitivity. Figure 13a shows the FM frequency responses for three devices (D1, D2, and D3) with different mass surfaces. D2 has a mass dimension of *l*_*m*_ = 1000 µm and *w*_*m*_ = 20 µm, while D3 has *l*_*m*_ = 1000 µm and *w*_*m*_ = 200 µm. D1 has the dimension of the proposed sensing device. It is clear that the resonance frequency values for D1 and D2 are almost the same since they have a mass surface of 25,000 µm^2^ and 20,000 µm^2^, respectively, Fig. 13a. Obviously, increasing mass surface (D3) decreases the resonance frequency. Figure 13b presents the amplitude transient results for those three devices of He gas sensing. The results show a small shifting in the response and recovery time. It is obvious that a small mass surface means a lower sensitivity. Figure 13c shows the sensitivity value for the three devices. Clearly, a big mass surface results in enhancing the sensitivity of the sensor. However, it was noticed that the response did not reach to the baseline during recovery process initiating the linearity and drift issues. Further investigations are required to fully understand the behavior of D2 and D3. The insets of Fig. 13c show the microscopic images of the devices.

**Figure 14:**
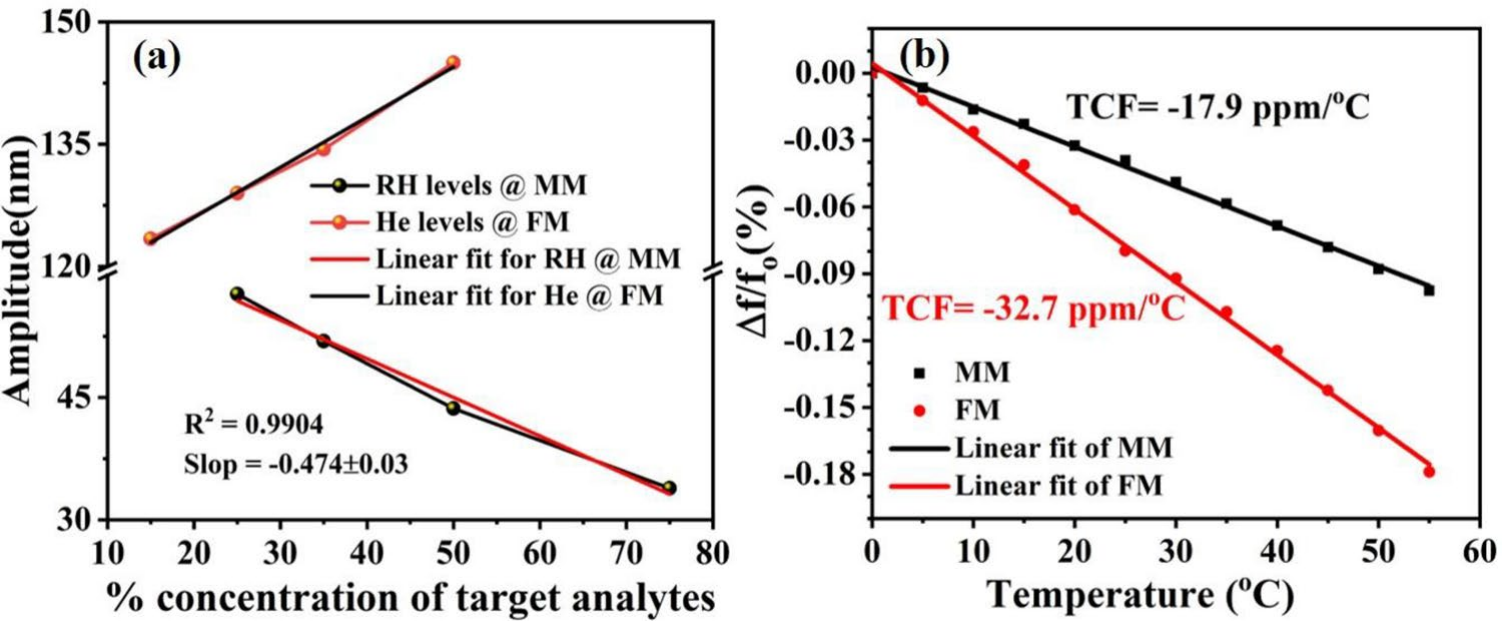
(**a**) The experimental and calculated linear fit results for different levels of RH at MM and He at FM (simultaneous actuation), and (**b**) simulated temperature dependence of frequency (TCF) of the device for both modes.

The experimental and calculated linear fit curves for different relative humidity and He concentrations levels at both modes are displayed in Fig. 14a. The sensor demonstrates nonlinearity of less than 1%. The performance of the proposed micro-sensor using individually and simultaneously actuated vibration modes are summarized in Table 1. Furthermore, the resonance frequency of the proposed sensor needs to be stable and immune from the environmental changes, such as temperature, which may have considerable effect on the device performance. Figure 14b shows the simulated variation of the resonance frequency of the moveable mass mode and the flexural beam mode versus temperature range from 10 to 55 °C. The simulated thermal coefficient of frequency (TCF) of the device for the two modes is found to be − 17.9 ppm/°C and − 32.7 ppm/°C, respectively. These values are obtained from the slope of the curve fit to the data in Fig. 13. These results indicate that the proposed silicon MEMS sensor has low sensitivity to the temperature variation, which is determined by the dependence on temperature of the mechanical and electrical properties of silicon, such as Young’s modulus, the resistivity, thermal expansion coefficient, and thermal conductivity. Table 2 shows a summary of the performance comparisons of the proposed micro-sensor with previously published MEMS based gas sensors. As can be seen that our proposed device demonstrate much stronger response to RH and He when compared to the previously published result^34,49,50^. Featured with low-cost, small size, and high performances such as high sensitivity, good linearity, and low power consumption, the proposed muti-mode sensor can be useful in the Internet of Things (IoT) and Wireless Sensor Network (WSN) applications.

**Table 1:** Summary of sensing results obtained with individually and simultaneously actuated vibration modes.

Type	Input power	Mode	Sensing material	Target analyte	Response/recovery time	Sensitivity	Non-linearity	Selectivity
Individual mode actuation	2.32 mW	MM	Graphene oxide	50% RH	200 s/450 s	0.47 nm/%RH	< 1% (70–25% RH)	Excellent to RH
				50% He	200 s/160 s	0.08 nm/%He		
		FM	Heated Si micro-beams	50% RH	110 s/120 s	0.08 nm/%RH		Excellent to He
				50% He	70 s/95 s	0.67 nm/%He		
Simultaneous mode actuation	3.16 mW	MM	Graphene oxide	50% RH	100 s/190 s	0.69 nm/%RH	< 1% (75–25% RH)	Excellent to RH
				50% He	90 s/100 s	0.57 nm/%He		
		FM	Heated Si micro-beams	50% RH	100 s/190 s	0.1 nm/%RH	< 1% (50–15% He)	Symmetric to He
				50% He	90 s/100 s	0.57 nm/%He

**Table 2:** Summary of the performance comparisons of the proposed sensor with previosuly published MEMS based gas sensors.

Device	Material	Response/recovery time	Sensitivity (S)	References
Piezoresistive micro-cantilever (electro-thermal)	ZnO NRs/Chitosan	57 s/–	~ 51 ppm^a^ /%RH	^34^
Piezoresistive micro-cantilever (electro-thermal)	Chitosan@ZnO NRs/Silicon	46 s/167 s	~ 16.9^a^ (ppm/%RH)	^49^
Micro-cantilever beam (external actuator)	Metal coated hydrogel	–	~ 650^a^ ppm/%RH	^50^
Micro-beams resonators (electromagnetic)	GO/n-doped Silicon (individually actuated)	200 s/450 s (50% RH)	~ 9890.1^a^ (ppm/%RH) @ MM	This work
		70 s/95 s (50% He)	7287.2^a^ (ppm/%He) @ FM	
	GO/n-doped Silicon (simultaneously actuated)	100 s/190 s	8523^a^ (ppm/%RH) @ MM	
		90 s/100 s	5845^a^ (ppm/%RH) @ FM	

^a^*S* (ppm) = $\frac{\Delta f or \Delta A}{{f}_{o }or {A}_{o}\times \Delta \% target analyte}\times {10}^{6}$ where $\Delta f$ and $\Delta A$ is change in frequency and amplitude after gas exposure,${f}_{o}$ and ${A}_{o}$ is baseline frequency and amplitude.

We should mention that in case of a mixed gas with three gases or more, the proposed technique does not lead to fully selective detection technique, and thus, it is not possible to discriminate the nature of the tested gases. Hence, to address the cross-sensitivity in mixed gas sensing, one of the solution is based on using data processing or machine learning technique^18,19^.

### Discussion on the physisorption sensing mechanism

**Figure 15:**
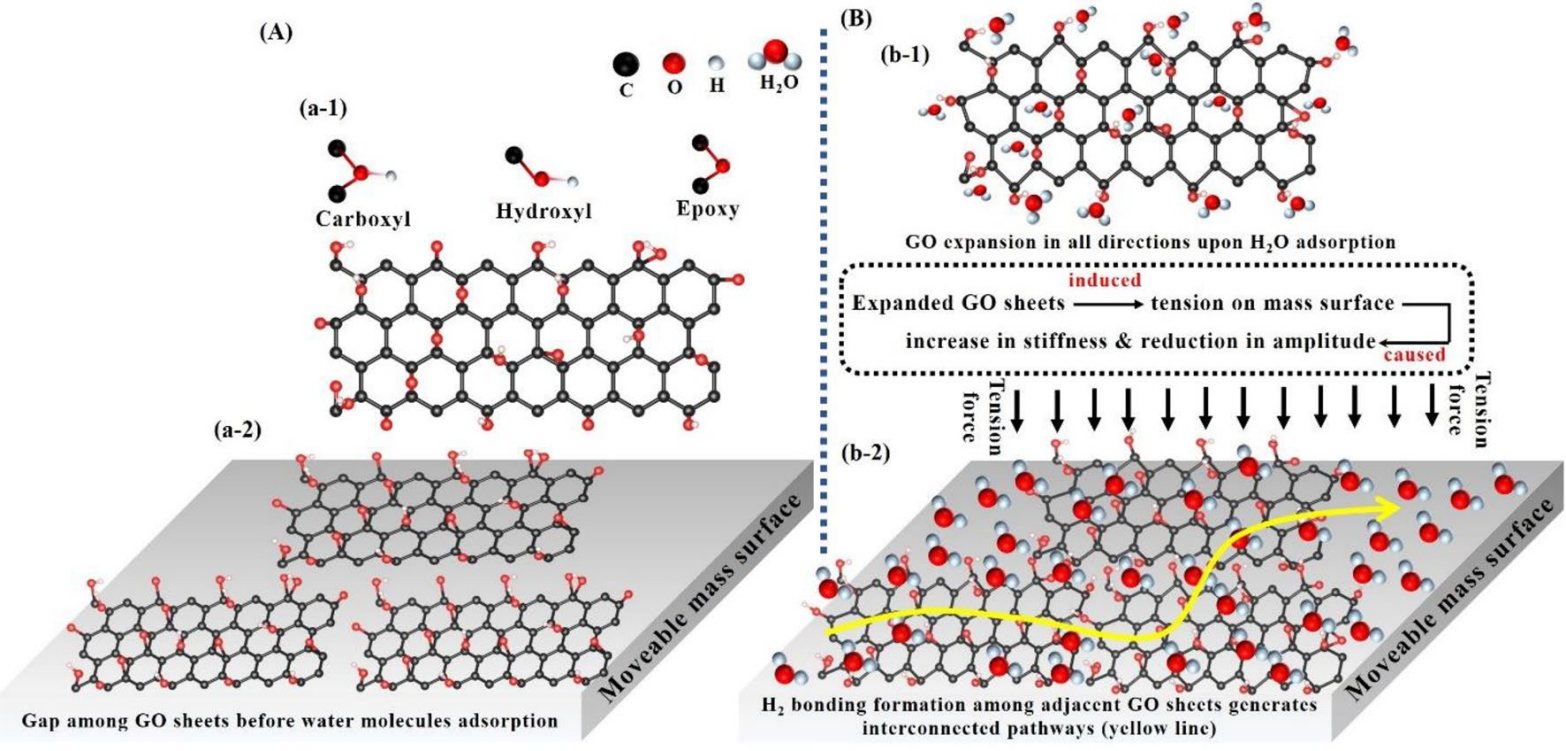
Sensing mechanism of water molecule adsorption on GO at the mass surface, (**A**) a schematic of GO sheets on moveable mass before water molecules exposure, (a-1) the front view of GO sheet with different functional groups attached on its surface (epoxy, hydroxyl, and carboxyl), (a-2) GO sheets on the moveable mass surface which indicates a gap among adjacent GO in the absence of water molecules, (**B**) a schematic of GO sheets on moveable mass after water molecules exposure, (b-1) illustrates the expansion of the GO sheet in all directions when water molecules form a bond with oxygen functional groups, (b-2) demonstrates the expansion of GO and development of H_2_ bonding with adjacent sheets to form interconnected GO sheets pathways, which induced a downward tension force and eventually lead to a decrease in amplitude and increase in frequency values at the mass mode.

Graphene and its derivatives (reduced graphene oxide and graphene oxide) have gained huge attention in several fields including, energy storage/super capacitors^45^, energy harvesting^46^, filtering/separation membrane^16^, and gas sensing due to its high surface area, high electron mobility at room temperature, porous structure, and excellent mechanical/thermal stability^47,48^. Among them, reduced graphene oxide (rGO) and graphene oxide (GO) hold great potential for sensing due to the existence of different functional groups on their surface. Particularly, GO is considered as one of the best-known materials for water vapors detection due to the existence of a large number of oxygen functional groups on its surface and edges^44,47^. For our study, the electrically insulated GO droplets were deposited onto a highly conductive Si resonator surface and dried at 45 °C for 30–45 min followed by direct sensing characterizations without further annealing. No barrier junction formation between electrically insulated GO and highly conductive Si surface can be realized under these conditions. Therefore, upon water vapors exposure the change in the electronics/electrical state of the GO may not significantly contribute to determining the sensing mechanism. On the contrary, it can be better explained through water vapor-induced deformation in GO, which builds an axial force on the cantilever mass beam and thus changes its mechanical behavior. It is well documented that the adsorption of water molecules leads to the expansion of graphene oxide (GO) by establishing hydrogen bonding with the functional groups of the GO layers^47,48^. It is believed that during water vapor exposure GO expands in all directions alongside the beam and might build bonding with adjacent sheets and form a chain of interconnected GO sheets, which induced a downward tension on the resonator as illustrated in Fig. 15. As a result, a decrease in amplitude and a small shift to higher frequency were observed in the mass mode. The higher sensitivity of the device can be attributed to the higher surface area of GO sheets, which provide more active sites for water vapors' adsorption.

## Conclusions

We investigated the multimode sensing behavior of a MEMS resonator composed of a clamped-guided arch beam connected to flexural beams and a T-shaped moveable mass for the selective detection of relative humidity and He gas. The sensor was tested when the vibration modes were individually and simultaneously actuated to observe the real-time change on both modes. It is found that the mass mode shows a strong response towards relative humidity with excellent selectivity while the axially actuated flexural beam shows a selective response to He gas. The excellent selectivity and sensitivity of the mass mode towards relative humidity can be attributed to the hydrophilic nature of GO. On the other hand, faster sensitivity and excellent selectivity of flexural mode to He can be attributed to the axially stressed micro-beam sensitivity towards the temperature change. Finally, we believe that the utilization of different vibration modes of a carefully designed MEMS device is a promising approach and may open up new frontiers for the development of smart and highly selective gas sensors.
